# Monitoring and modelling the glutamine metabolic pathway: a review and future perspectives

**DOI:** 10.1007/s11306-023-02031-9

**Published:** 2023-07-23

**Authors:** Zohreh Mirveis, Orla Howe, Paul Cahill, Nitin Patil, Hugh J. Byrne

**Affiliations:** 1 FOCAS Research Institute, Technological University Dublin, City Campus, Camden Row, Dublin 8, Ireland; 2 School of Physics and Optometric & Clinical Sciences, Technological University Dublin, City Campus, Grangegorman, Dublin 7, Ireland; 3 School of Biological, Health and Sport Sciences, Technological University Dublin, City Campus, Grangegorman, Dublin 7, Ireland; 4 School of Biotechnology, Dublin City University, Glasnevin, Dublin 9, Ireland

**Keywords:** Metabolic pathways analysis, Glutaminolysis, Flux analysis, Computational modelling, Vibrational spectroscopy, Chemometrics

## Abstract

**Background:**

Analysis of the glutamine metabolic pathway has taken a special place in metabolomics research in recent years, given its important role in cell biosynthesis and bioenergetics across several disorders, especially in cancer cell survival. The science of metabolomics addresses the intricate intracellular metabolic network by exploring and understanding how cells function and respond to external or internal perturbations to identify potential therapeutic targets. However, despite recent advances in metabolomics, monitoring the kinetics of a metabolic pathway in a living cell in situ*,* real-time and holistically remains a significant challenge.

**Aim:**

This review paper explores the range of analytical approaches for monitoring metabolic pathways, as well as physicochemical modeling techniques, with a focus on glutamine metabolism. We discuss the advantages and disadvantages of each method and explore the potential of label-free Raman microspectroscopy, in conjunction with kinetic modeling, to enable real-time and in situ monitoring of the cellular kinetics of the glutamine metabolic pathway.

**Key scientific concepts:**

Given its important role in cell metabolism, the ability to monitor and model the glutamine metabolic pathways are highlighted. Novel, label free approaches have the potential to revolutionise metabolic biosensing, laying the foundation for a new paradigm in metabolomics research and addressing the challenges in monitoring metabolic pathways in living cells.

**Supplementary Information:**

The online version of this article (10.1007/s11306-023-02031-9) contains supplementary material, which is available to authorized users.

## Introduction

Central carbon metabolism is critical for energy production and includes the glycolysis pathway, the pentose phosphate pathway and the tricarboxylic acid (TCA) cycle. Increased understanding has revealed that the roles of these metabolic pathways are not limited to cellular energetics, but they are also involved in the assembly of the most important cellular building blocks, including nucleotides and amino acids (Zhang et al., 2008). Concomitant with the advances in knowledge and understanding of this metabolic system, a different view of glutamine metabolism has emerged (Reitzer et al., 1979), one that elevates glutamine from a non-essential amino acid to one which plays a fundamental role in cell biosynthesis and bioenergetics, including redox homeostasis, synthesis of metabolites that drive the TCA cycle, generation of antioxidants to eliminate reactive oxygen species (ROS), synthesis of nonessential amino acids, pyrimidines, purines, and macromolecules such as fatty acids for cell replication and activation of cell signaling (Deberardinis & Cheng, 2009; Newsholme et al., 2003). Moreover, glutamine has been shown to be a perfect substrate for oxidative metabolism in glutamine-addicted tumour cells (Wise & Thompson, 2010). These properties make glutamine metabolism an attractive target for therapeutic interventions designed to effectively disrupt cancer cell metabolism (Altman et al., 2016; Matés et al., 2020). Therefore, monitoring and elucidating intracellular glutamine metabolism as a potential therapeutic target, especially in cancer, has attracted the interest of researchers in recent decades (Zhu et al., 2017).

The science of metabolomics is a rapidly growing field of research that focuses on the identification and quantification of metabolites present in a biological system, such as cells or tissues. This field aims to identify and elucidate active metabolic pathways in a given system and to understand how these pathways are affected by different conditions or stimuli. Although there are several approaches to metabolomics research, the primary focus has historically been on the intracellular metabolome, mainly aimed at exploring cellular metabolism to elucidate its functional mechanisms and responses, which is beneficial not only to disease diagnosis but also to many aspects of toxicology and therapeutics (Doroghazi et al., 2014; Guma et al., 2016; Wishart, 2016). In the context of glutamine metabolism, metabolomics can be used to identify the metabolites that are produced or consumed during the breakdown and synthesis of glutamine, and to study the effects of various genetic or environmental factors on this process.

While metabolomics focuses on identifying and quantifying the metabolites, fluxomics has emerged as the process of studying metabolic fluxes, or the rates of biochemical reactions, in a biological system, which is a more dynamic and quantitative approach to understanding metabolism (Emwas et al., 2022). Metabolic fluxes are altered in many diseases, such as cancer, and therefore elucidating how the rate of metabolic processes changes can be of great benefit for the understanding of risk factors, disease development and progression, and development of therapies (Antoniewicz, 2018). Fluxomics approaches such as metabolic flux analysis (MFA) and flux balance analysis (FBA) have advanced various steady-state and kinetic approaches to monitor and analyse metabolic fluxes, which have accelerated the understanding of metabolism (Yasemi & Jolicoeur, 2021). Physicochemical models can be developed to provide insights into how the properties of biological systems come about and how their behaviour can be predicted and regulated (Bordbar et al., 2014; Khodayari et al., 2014). Metabolic network analysis (data-driven approach) based on pseudo steady state (PSS) and dynamic assumptions have been combined with physicochemical models to capture the dynamic behaviour of biological samples (Antoniewicz, 2013a).

**Fig. 1:**
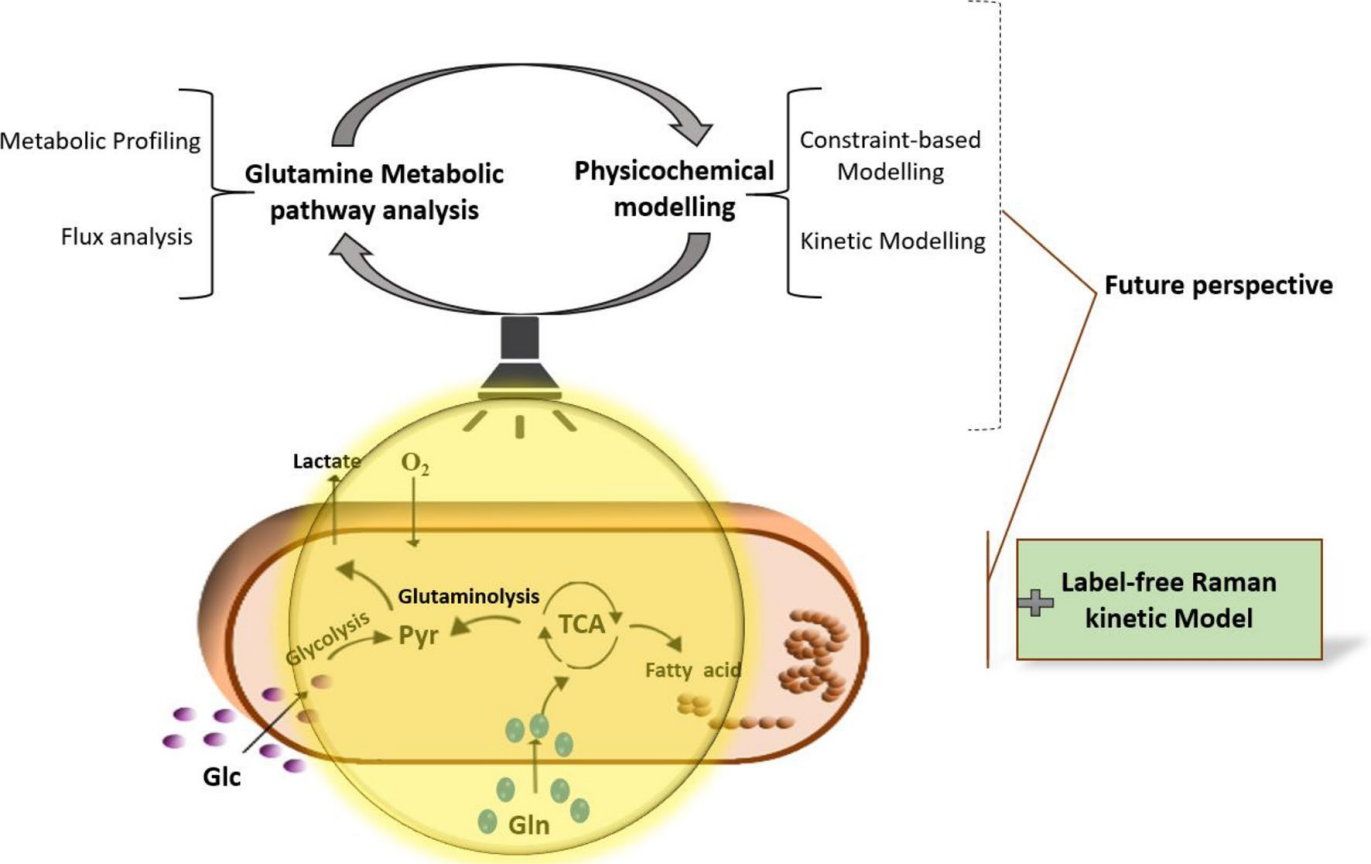
Combining analytical approaches, including fluxomics techniques and profiling tools, with physicochemical modelling approaches helps to elucidate a metabolic pathway such as glutamine and provide a deep understanding of cellular function and responses

This review paper (as summarised in Fig. 1) explores various analytical approaches used to investigate intracellular metabolism, including metabolic profiling methods [typically using mass spectrometry (MS) and nuclear magnetic resonance (NMR)] and flux analysis techniques (FBA and MFA). Additionally, we discuss two types of physicochemical modeling techniques commonly used in metabolomics and how they have helped to consolidate and expand our understanding of metabolic processes. Using glutamine metabolisation as an exemplar, the limitations and challenges associated with these techniques, as well as their advantages and insights, are explored.

The lack of a technique that can monitor the kinetics of a metabolic pathway in a living cell in situ is identified as a challenge. To address this limitation, we investigate the potential of label-free vibrational spectroscopy techniques, such as Raman microspectroscopy, which can enable real-time and in situ monitoring of the cellular kinetics of metabolic pathways with subcellular resolution.

In the “Future Perspectives” section, we suggest a combinatorial approach to address the current limitations, which involves utilizing label-free Raman microspectroscopy along with kinetic models trained by the biochemical assays, to enable real-time and in situ monitoring of the cellular kinetics of the glutamine metabolic pathway. While vibrational microspectroscopy is not expected to replace metabolomics, it can potentially complement it by operating in the niche areas where metabolomics falls short. Vibrational spectroscopy can provide the spectral signatures of the cellular complexities, but it cannot distinguish between different metabolites, which demands innovative data analysis tools to extract the dynamics of cellular metabolism, which will be discussed in more detail in the last section.

## Glutamine metabolism

**Fig. 2:**
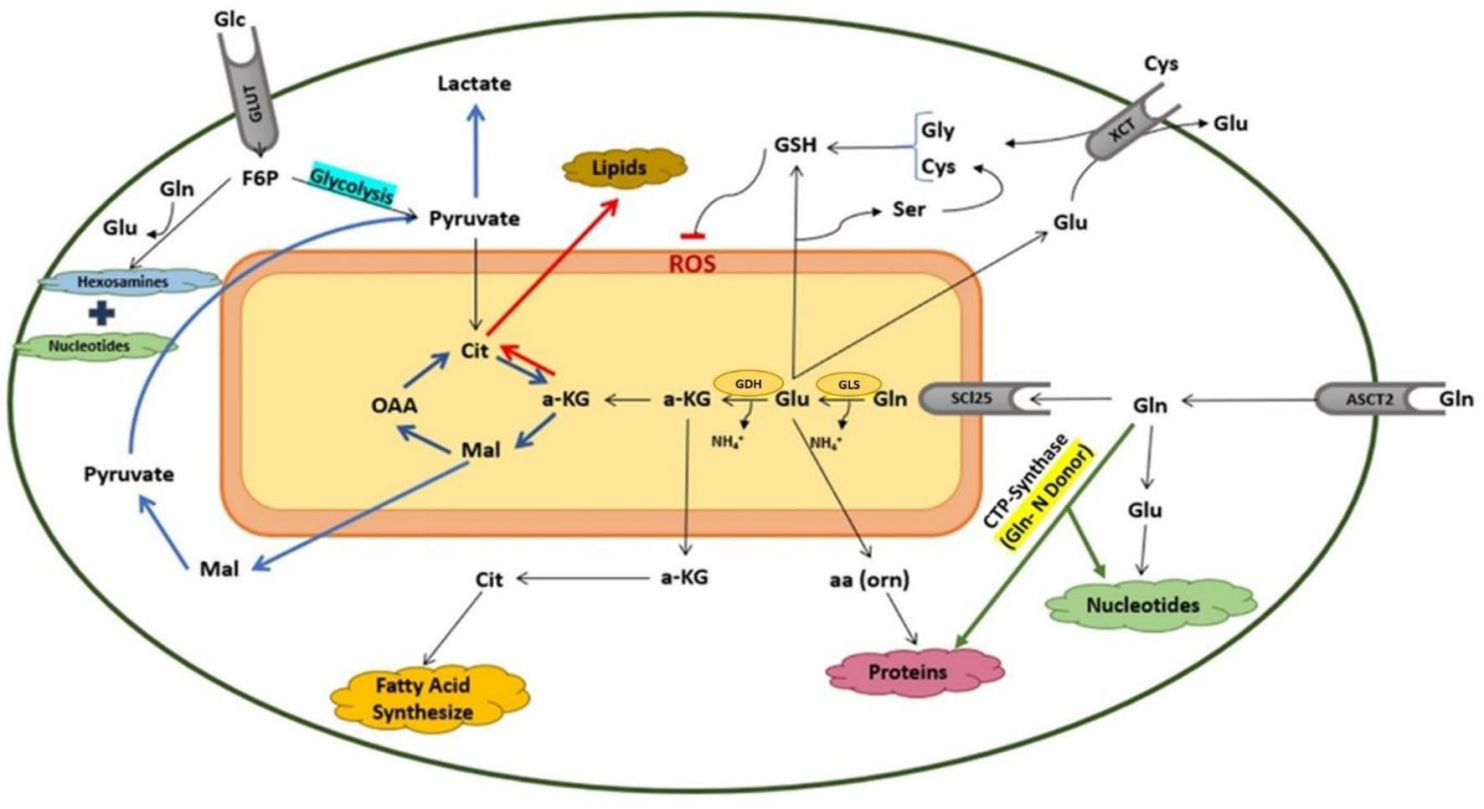
Schematic summary of intracellular glutamine metabolism. Glutamine is involved in the synthesis of four main macromolecules in the cytosol: proteins, nucleotides, lipids and fatty acids. In mitochondria, alpha-ketoglutarate derived from glutamine can be further metabolised either by oxidation involving the TCA cycle, termed as the glutaminolysis pathway (indicated by blue arrows), leading to lactate production, or by the reductive carboxylation pathway (indicated by red arrows), leading to lipid synthesis. *Gln* glutamine, *Glc* glucose, *Glu* glutamate, *α-KG α*-ketoglutarate, *Mal* malate, *OAA* oxaloacetate, *Cit* citrate, *GSH* glutathione, *aa* acid amine, *Gly* glycine, *Ser* serine, *Cys* cysteine (Color figure online)

Extracellular glutamine is predominantly internalised by cells via the alanine-serine-cysteine transporter 2 (ASCT2), which has itself been the target of many studies, because it is overexpressed in various types of cancer cells (Liu et al., 2018a), including breast cancer, lung cancer and liver cancer (Liu et al., 2018b). Glutamine plays diverse roles in cells, depending on the pathway by which it is metabolised. Figure 2 summarises the main contributions of glutamine to cellular metabolism. After entering the mitochondria, glutamine is converted to glutamate by the enzyme glutaminase (GLS). Subsequently, glutamate undergoes either oxidative deamination by glutamate dehydrogenase (GDH) in the mitochondria, leading to the generation of α-ketoglutarate (α-KG), or transamination in the cytosol, resulting in the production of non-essential amino acids (Li & Le, 2018; Newsholme et al., 2003). α-KG can follow two pathways. In the first, it undergoes oxidation within the TCA cycle [often called the Szent-Györgyi–Krebs cycle, acknowledging the Nobel Prize (1937) winning contributions of Albert Szent-Györgyi in identifying the fumarate, malate and oxaloacetate forming reactions towards the end of the cycle, and those of Hans Adolf Krebs, Nobel Prize (1953), for elucidating the details of the overall cycle], ultimately being converted to pyruvate and lactate in the cytosol through a metabolic process known as glutaminolysis (represented by blue arrows) (‘Albert Szent-Györgyi – Facts—NobelPrize.org’ n.d.; Altman et al., 2016; Krebs & Johnson, 1980; Wang et al., 2010). This process not only drives the TCA cycle and fuels the electron transport chain (ETC), a major energy source, but also participates in supporting redox homeostasis by supplying carbon to the malic enzyme, certain isoforms of which generate NADPH required for anabolic processes such as fatty acid synthesis (Lyssiotis et al., 2013). In the second pathway (represented by red arrows), α-KG undergoes reductive carboxylation, contributing to the synthesis of citrate in the TCA cycle (Yang et al., 2017a), which then contributes in lipid biosynthesis while maintaining the redox balance within the mitochondria (Mullen et al., 2014).

Glutamine also plays a role in promoting the synthesis of macromolecules such as fatty acids, lipids, proteins, and nucleotides by providing carbon and nitrogen for their precursors (DeBerardinis et al., 2007). Additionally, it affects the activity of cytokines such as Tumour Necrosis Factor-alpha (TNF-a) and Interleukin-6 (IL-6) and other factors that boost the immune system (Cruzat et al., 2018). Furthermore, glutamine participates in the production of the antioxidant glutathione (GSH), inhibiting ROS and supporting various biosynthetic pathways crucial for cellular integrity and function (Amores-Sánchez & Medina, 1999).

Abnormalities in glutamate/glutamine metabolism have been linked to disorders such as hyperinsulinism/hyperammonaemia syndrome (Kelly & Stanley, 2001) and mental health disorders like bipolar disorder (Moore et al., 2007) and depression (Abdallah et al., 2014). Moreover, glutamine can be targeted therapeutically, as demonstrated by its ability to repolarize macrophages from an M1 to an M2 phenotype, which can prevent obesity- or diabetes-associated pathology (Ren et al., 2019). Moreover, glutamine metabolism is a crucial topic in cancer research. The Warburg effect, observed in tumor cells, involves the conversion of glycolytic pyruvate to lactate instead of further catabolism through the TCA cycle, leading to reduced energy production per mole of glucose (Manoj et al., 2022; Warburg, 1956). Glutaminolysis serves as an alternative energy source in tumor cells, and its acceleration has been observed in glutamine-addicted cancers such as highly invasive ovarian and mesothelioma cancers (Adhikary et al., 2022; Medina, 2001; Li & Le, 2018; Yang et al., 2017a). However, the role of glutamine in continuous cell proliferation as an anabolic substrate is controversial, as its limited availability in tissues and circulation restricts its biological role in this process (Brosnan, 2003; Lacey & Wilmore, 1990). Glutamine is not replenished upon exhaustion, nor is it produced in sufficient amounts during stress or uncontrolled cancer growth. Therefore, the body must obtain glutamine from the diet during high demands, making it a conditionally essential amino acid (Lacey & Wilmore, 1990). As a result, glutamine can only be considered a conditionally indispensable substrate and/or precursor in the human circulation with limited direct roles as a carbon source in the proliferation and growth of transformed cells.

Apart from its supportive role as a survival factor in the proliferation of cancer cells, glutamine is also involved in cancer suppression through several mechanisms. These include stimulating the activation of the tumour suppressor p53, which causes apoptosis and tumour regression (Suzuki et al., 2010). The expression levels of oncogenes and tumour suppressors have been found to be the most important factors determining the function of glutamine in cancer development or suppression (Iurlaro et al., 2014; Yoo et al., 2020). Therefore, monitoring the glutamine metabolism in a dynamic sense can be valuable for understanding cancer mechanisms and developing novel personalised therapies.

## Monitoring the glutamine metabolic pathway

The cellular metabolome accurately reflects the cellular phenotype and function (Beger, 2013), and in vitro metabolomic analysis has played a crucial role in advancing our fundamental understanding of cellular function. However, it is crucial to confirm the results in vivo, as some studies have indicated a significant mismatch between in vivo and in vitro measurements. For example, microglia immunometabolism exhibits a distinct phenotype in vivo compared to primary microglia cultures due to microglia's ability to adapt their energy metabolism to fluctuating nutrient availability (Bernier et al., 2020). Also, while glutamine serves as an anaplerotic substrate in the TCA cycle in glioma cells (DeBerardinis et al., 2007), tracking of ^13^C-Gln in brain tumor patients has shown minimal contribution of glutamine in the TCA cycle (Marin-Valencia et al., 2012; Mashimo et al., 2014). In this context, monitoring the glutamine metabolic pathway requires both in vitro and in vivo assessment to obtain reliable results. Advanced metabolic imaging techniques such as magnetic resonance spectroscopy (MRS), positron emission tomography (PET), single photon emission computed tomography (SPECT), MS imaging (MSI), and fluorescence imaging enable in vivo evaluation of glutamine metabolism for clinical diagnostics (Ekici et al., 2022). These techniques have already been described and explored in detail in the review by Ekici et al. (2022).

**Fig. 3:**
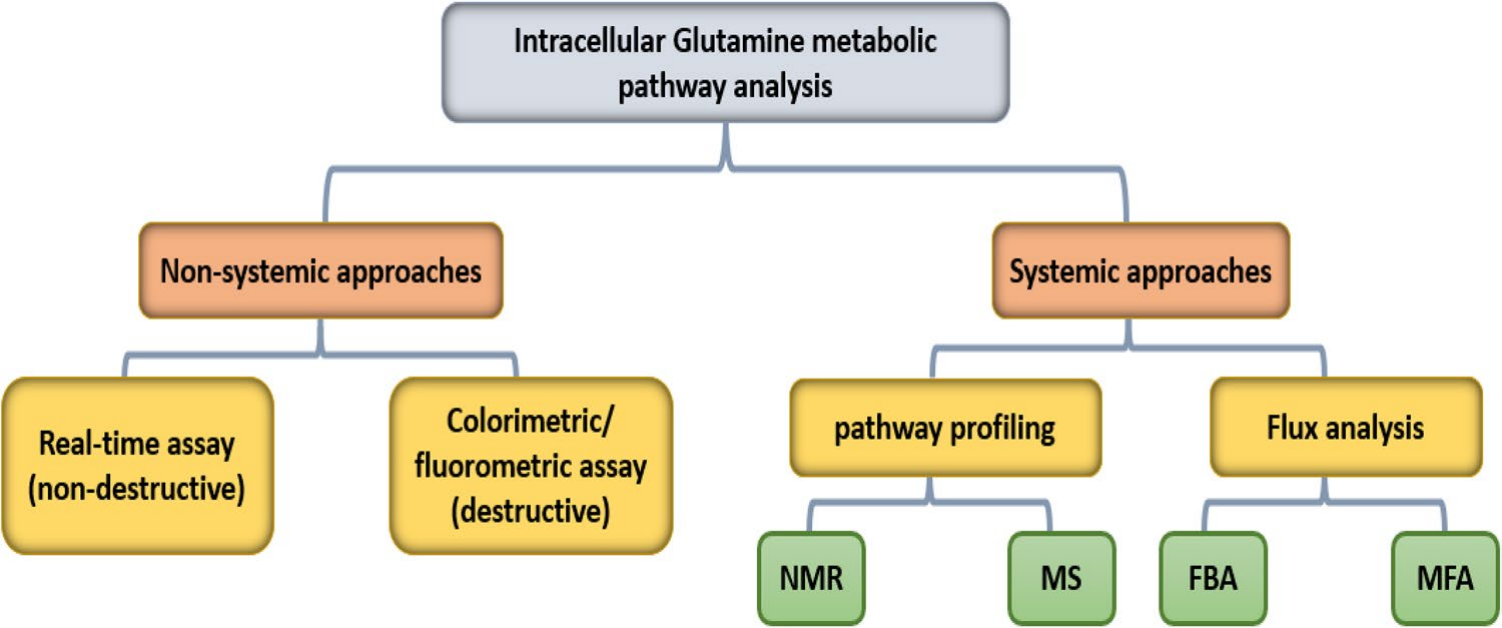
Simplified summary of methods for monitoring the glutamine metabolic pathway

This paper focusses on evaluating the available tools and techniques for monitoring the intracellular glutamine metabolic pathway in in vitro studies. To organize the techniques, they have been grouped into two categories, namely systemic and non-systemic approaches, as illustrated in Fig. 3.

### Systemic monitoring of the glutamine metabolism by metabolomics

Metabolomics is considered a powerful strategy to gain a systems-level understanding of metabolism and its changes in response to a specific perturbation in biological systems (Ortmayr et al., 2016a, b). This approach works in both a targeted and non-targeted mode, depending on the objective of the study. Metabolomics is treated as a relative quantification technique primarily aimed at obtaining accurate metabolic fingerprints for the maximum number of metabolites detected and subsequently identified in cells (Ortmayr et al., 2016a, b). This systemic metabolomic approach not only measures representative metabolites originating from different metabolic pathways, but can also reveal unknown metabolic pathways and their potential interactions. It thus avoids the limitation of hypothesis-driven methods (in systemic and non-systemic studies), which can lead researchers to overlook the effect of therapy on the entire cellular metabolism (Di Minno et al., 2019; Ribbenstedt et al., 2018; Zhang et al., 2016). Strategies used to holistically study glutamine metabolism include (1) metabolic profiling and, (2) analysis of metabolic activity (fluxes) using stable isotope labelling methods, mostly based on mass spectroscopy (MS) and NMR.

#### Metabolic profiling

Metabolic profiling approaches allow quantification of hundreds of metabolites in a biological cell, tissue, or organism (Kopka et al., 2004). They provide a more holistic picture of the state of cellular metabolism by providing large-scale detection and information on the structural composition and abundance of a broad range of metabolites, although it must be noted it is not possible to detect every metabolite in a system (Amantonico et al., 2010; Kopka et al., 2004). Untargeted profiling of the metabolome is performed using label-free analytical techniques, MS and NMR-based approaches being the most popular (Kuehnbaum & Britz-Mckibbin, 2013).

##### Mass spectrometry

The mass spectrometer ionises the chemical components and sorts the ions based on their mass-to-charge ratio (Han & Gross, 2005). The application of MS-based analytical approaches to the field of metabolomics studies has been motivated primarily by the recognised advantages in terms of specificity, sensitivity and a broad detection range that ensure powerful identification and quantification (Theodoridis et al., 2012). An example of its application in the context of glutamine metabolism is the work of Zhou et al. (2012), who elucidated a previously unknown mechanism of glutamine metabolism through a comparative MS analysis of the pancreatic ductal adenocarcinoma cell line. This revealed significant consumption of glutamine as a nitrogen donor in amino acid and nucleotide synthesis (shown by green arrows in Fig. 2), based on the observation of a strong overexpression of the enzyme cytidine triphosphate (CTP) synthase, which catalyses glutamine for the biosynthesis of cytidine, and down-regulation of TCA cycle's enzymes (Zhou et al., 2012).

A serious problem of MS in the identification of metabolites is ion suppression, which is a kind of matrix effect and is mainly caused by the different volatility of metabolites, due to their different nature and structure, which in turn affects the output of the number of ions in the gas phase reaching the detector and eventually leads to errors in the quantification of the analysis (Annesley, 2003; Antignac et al., 2005; Bamba et al., 2012). To address the issue of ion suppression and improve the identification of metabolites in MS analysis, chromatographic separation techniques such as gas chromatography (GC) and liquid chromatography (LC) have been employed (Sandra & Sandra, 2006). These techniques can effectively separate metabolites from interfering matrix compounds in the sample, based on their relative mobilities in the stationary phase which results in better resolution and more accurate quantification of metabolites (Kole et al., 2011). In the flux analysis approach, which will be explored Sect. 3.1.2, the measurement of different isotopomers requires the measurement of different masses, but when it comes to measuring different metabolites, chromatographic separation is preferred to ensure accurate identification and quantification. Moreover, the integration of GC–MS and LC–MS techniques offers a broader coverage of metabolites and facilitates a more comprehensive understanding of complex biological systems in metabolomics studies. Zeki et al. (2020), conducted a comprehensive review highlighting the simultaneous utilisation of GC–MS and LC–MS in untargeted metabolomics investigations, highlighting crucial aspects such as sample preparation, data acquisition, and data processing. Also, advancements in technology have led to the development of new techniques such as supercritical fluid chromatography (SFC), which shows high sensitivity comparable to or even better than LC–MS (Losacco et al., 2019) and technologies like matrix-assisted laser desorption ionization (MALDI) and desorption electrospray ionization (DESI) MS that allow for the spatial identification of metabolites (Matés et al., 2020). These advancements offer further opportunities for improved metabolite analysis.

Another major challenge is the reliability in identifying the detected metabolites. This problem has been solved with Molecular Networking, a visualisation method for tandem MS data (Hollywood et al., 2018). This method assumes that similar molecules have similar MS fragmentation patterns, meaning that they tend to group closely together in a network, and is able to detect the spectral sets of related molecules (Hollywood et al., 2018). Despite all the progress made in MS approaches, such as improving the detection limit to the sub-attomole to zeptomole range, there are still some limitations in using it at the single cell level (Liu et al., 2019).

##### Nuclear magnetic resonance spectroscopy

**Fig. 4:**
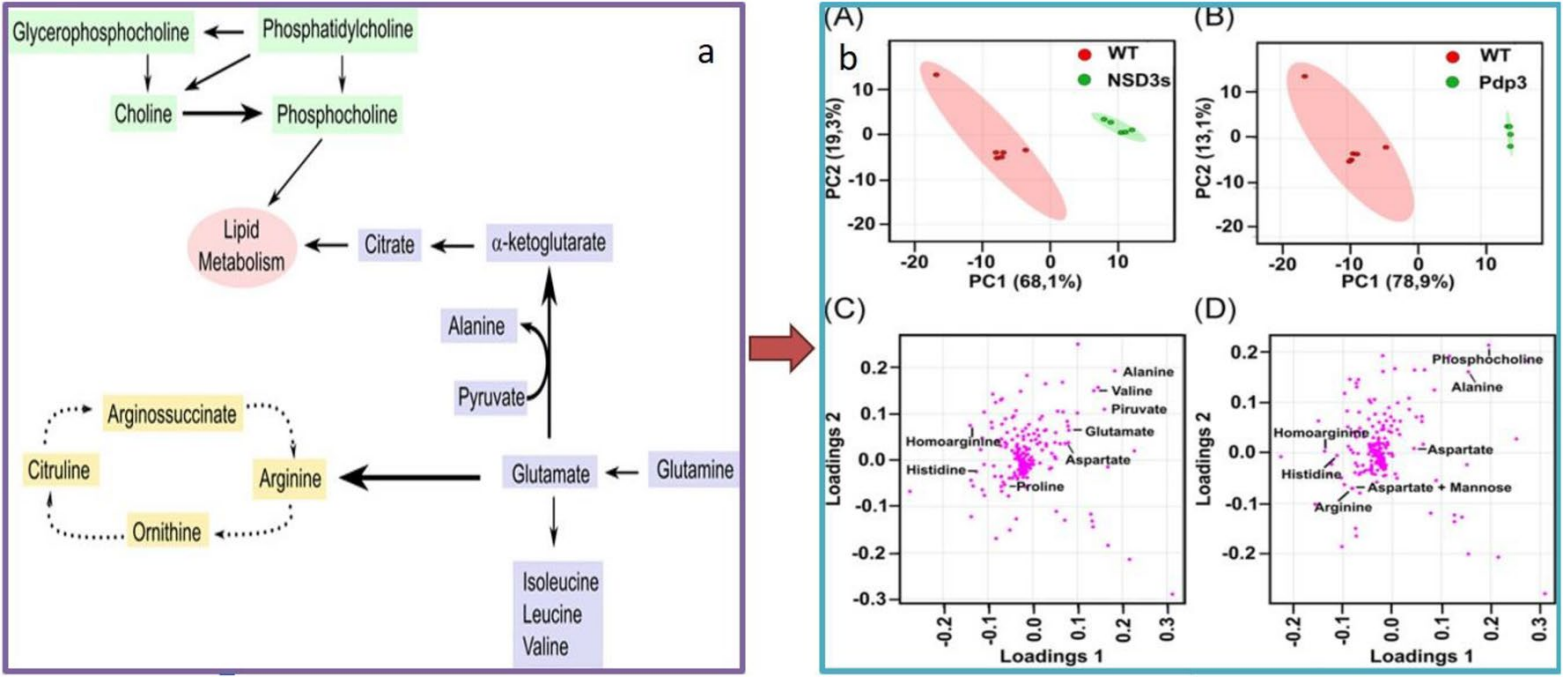
**a** Metabolic pathways of the major metabolites detected on NSD3s and Pdp3 overexpression. The main metabolites detected belong to glutaminolysis (blue), metabolism of cholines (green), and arginine synthesis (yellow). **b** NMR metabolomics shows similar metabolic profile on NSD3s or Pdp3 overexpression. PCA score plots show a strong class discrimination between control (WT) replicates (red) and NSD3s overexpression (green) (A), and between control (WT) replicates (red) and Pdp3 overexpression (green) (B). PCA loading plots highlight important metabolites for class discrimination between NSD3s+ and WT, such as glutamate, aspartate, and alanine (C), and between Pdp3+ and WT, such as phosphocholine, aspartate, and alanine (D). *NMR* nuclear magnetic resonance, *WT* wild-type. Reproduced from Rona et al. (2019) with permission

NMR is another high-throughput metabolomics technology that has been used in many studies to analyse metabolomics profiles (Reo, 2002). Recently, Lee et al. used ^1^H NMR spectroscopy to identify metabolic pathways that are disrupted in gliomas, including lactate, glutamine and alanine, to determine the metabolic characteristics of gliomas that are candidates for targeted therapies (Lee et al., 2019). Another recent study related to glutamine metabolism using NMR revealed a link between the human oncoprotein NSD3s and the rewiring of cancer metabolism by taking the glutaminolysis pathway (Fig. 4a). This study analysed the metabolic response to overexpression of NSD3s in *Saccharomyces cerevisiae* using ^1^H NMR and reported that overexpression leads to a simultaneous increase in aspartate and alanine levels and a decrease in arginine levels, which in turn leads to an increase in the rate of glutaminolysis (Fig. 4b) (Rona et al., 2019). Some other applications of NMR as a metabolic profiling tool have been discussed in the work of Larive et al. (2015).

On the other hand, analytical instruments for metabolic studies on living cells must not only provide high-resolution data, but also be rapid, since the physiological state of the living cell can change for instance, if it experiences oxygen deprivation for more than a few minutes (Serber et al., 2007). Monitoring the metabolism of living cells by 1D and 2D NMR spectroscopy is limited by the low resolution of the spectra and the long time required, respectively (Motta et al., 2003). Some efforts have been made to solve this issue, such as the application of band-selective optimised flip-angle short-transient (SOFAST) HMQC techniques to ^15^N-labelled cells, which provide high-resolution spectra of small metabolites directly in living cells in a few seconds (Motta et al., 2003). Although NMR using fluorescent biosensors or optical reporter tags can provide a valuable perspective for identifying the biochemistry of living cells, it, like the MS based approach, is of limited use for in situ tracking of metabolic processes (Lerche et al., 2015), due to the inherent physicochemical properties of small and hydrophilic metabolites, such as low ionisation efficiency and high detection limits (Chalcraft et al., 2009; Kuehnbaum & Britz-Mckibbin, 2013). Furthermore, the datasets generated by NMR and MS are complicated and difficult to model and interpret, although progress continues to be made in statistical approaches to data mining (Coen et al., 2008).

#### Flux analysis of cellular metabolism

Flux analysis (fluxomics) techniques are used to study the rates of metabolic transitions that are critical for identifying key regulatory nodes in metabolic pathways, based on changes in rates under intra/extracellular perturbations. Understanding the metabolic dynamics and fluxes through these techniques serves as the foundation for comprehending disease mechanisms/phenotypes and developing novel therapies (Metallo & Vander Heiden, 2013; Wishart, 2016). Intracellular metabolic fluxes can be estimated through both MFAs and FBA (Toya et al., 2011). MFA is a technique used to quantify reaction fluxes in metabolic pathways over time, utilising the stable isotope labeling method. In this method, isotopic isomers, or isotopomers, are introduced into the biological system, and based on prior knowledge of carbon transitions through the metabolic pathways, fluxes through different nodes are calculated (Allen & Young, 2020; Yang et al., 2002). MFA estimates intracellular fluxes empirically from labelling patterns in metabolic networks to represent the in vivo metabolic state of the cell (Iwatani et al., 2008). The choice of tracer determines the accuracy of the estimation of metabolic fluxes, as it enables more targeted analysis to investigate specific reactions within the network (Metallo et al., 2009). For example, ^13^C-labelled glutamine and ^15^N-labelled glutamine can be used to track the fate of carbons and nitrogen, respectively, in glutamine metabolism (Yang et al., 2017b). There are two main experimental procedures for the estimation of metabolic fluxes by isotope tracers: dynamic (kinetic) and steady-state tracing (Emwas et al., 2022). In kinetic isotope labeling experiments, an unlabelled carbon source like glucose or glutamine is replaced by a labeled one, and the labelling patterns of intermediates and products are measured at different time points (Wang et al., 2020). Conversely, in steady-state experiments, the labelled material is distributed through metabolic pathways until a steady state of label accumulation is achieved in each intracellular metabolite pool (Buescher et al., 2015). Wang et al. provide a review of the development of equations used to determine reaction fluxes derived from labelling patterns applied to biological systems (Wang et al., 2020).

In the context of glutamine metabolic pathways, tracking the fate of labelled metabolites has established the contribution of glutamine carbon atoms to central carbon metabolism pathways, including glutaminolysis, the TCA cycle, and the pentose phosphate pathway (Leippe et al., 2017). Studies using ^13^C-Gln as an isotopic tracer have revealed that glutamine drives the TCA cycle, and its contribution increases significantly under hypoxia conditions, highlighting the key role of glutaminolysis metabolism in cancer cells (Zhang et al., 2014). Additionally, ^13^C-labelling studies have provided new insights into the reverse flux or reductive carboxylation of glutamine-derived α-KG through the TCA cycle (Kalyanaraman et al., 2018). In one example, Le et al. (2012) used the tracer [U-^13^C, ^15^N]-Gln to investigate the production rate of TCA cycle intermediates from glutamine in human lymphoma B cells (P493) under glucose depletion. The researchers found that P493 cells rely on a glutamine-driven TCA cycle independent of glucose and that inhibiting GLS curbs tumour cell proliferation both in vitro and in vivo. However, intervention or inhibition of the glutamine pathway does not always result in a therapeutically effective outcome such as apoptosis. Alternative metabolic pathways can be activated to compensate for the required energy deficiency (Lopez & Banerji, 2017; Thompson et al., 2017). Numerous studies have reported this, including that of Halama et al. (2018), which investigated the impact of inhibiting GLS C (GAC) in mouse embryonic fibroblasts and found that using the allosteric inhibitor C.968 had no effect on the expected decrease in glutamate and TCA cycle intermediates. These findings suggest that cancer cells may depend on other sources of energy, such as lipid catabolism and autophagy, which are known to accelerate in response to glutamate deficiency.

Isotopic labelling has become a popular approach for studying metabolic pathways, such as glutamine metabolism, and has provided valuable insights into metabolomics science. Nevertheless, there are some drawbacks that must be acknowledged. The primary limitation of isotopic labelling is related to the techniques used to measure isotopic labelling of molecules, such as NMR and MS. NMR spectroscopy is a powerful tool for understanding metabolic fluxes, including reaction rates and metabolite exchange between cell compartments, as well as providing positional labelling information (Tang et al., 2009; Yu et al., 1995). However, there are limitations such as time-consuming sample purification, lower sensitivity and resolution compared to MS, and expensive instrumentation (Lim et al., 2007). The recent review by Giraudeau highlights the ongoing efforts to improve NMR's sensitivity and resolution while maintaining its structure elucidation capabilities (Giraudeau, 2020). Additionally, the high complementarity between MS and NMR has been demonstrated in various studies, and the development of advanced statistical approaches for integrating data from multiple platforms shows promise (Giraudeau, 2020).

Compared to NMR, MS offers high analytical power, allowing accurate and rapid quantification of labelling patterns, making it more suitable for flux measurements due to its widespread availability, lower cost, and sensitive detection of molecular enrichment (Antoniewicz, 2013b; Goudar et al., 2010; Kleijn et al., 2007; Tang et al., 2009). However, MS analysis provides only partial information on the distribution of isotopomers, and it is challenging to extract information about the position of the label from MS data, even when multiple fragments of the same compound are detected (Kohlstedt & Wittmann, 2019). To address this limitation, Tandem-MS (MS/MS) can potentially increase the amount of labelling data available for MFA (Choi & Antoniewicz, 2011). Nonetheless, it remains a destructive technique that cannot monitor cellular kinetics in situ and can only provide a snapshot of the metabolic state at the time of analysis, which may not accurately reflect the dynamic nature of cells and their rapidly changing metabolic state (Wang et al., 2016).

Another limitation of isotopic labelling is that it can introduce potential biases in the metabolism due to the labeled carbon source being processed differently from the unlabelled carbon source, leading to misleading results (Bianco & Perrotta, 2015; Chaube, 2014; Hernandez-Saavedra et al., 2020). These limitations have prompted researchers to explore alternative, non-destructive methods, such as “label-free” techniques, which offer advantages such as low damage to cells, high sensitivity, and small working volumes (Cunningham & Laing, 2008; Krafft et al., 2017).

### Non-systemic simple biochemical assays

**Fig. 5:**
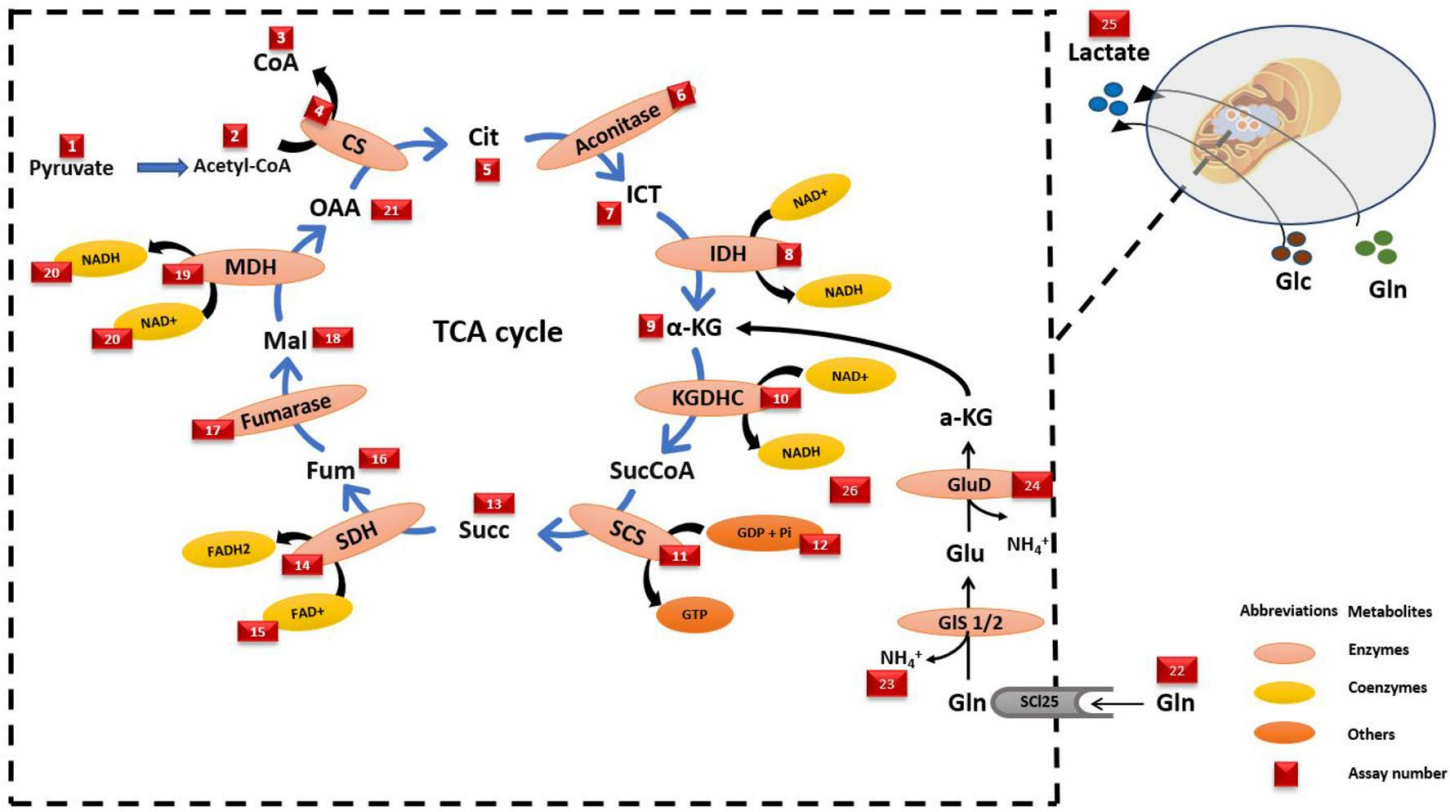
Depiction of the metabolites/enzymes involved in glutamine metabolism that can be measured by biochemical assays. *CoA* coenzyme-A, *Cit* citrate, *ICT* isocitrate, *IDH* isocitrate dehydrogenase, *α-KG α*-ketoglutarate, *KGDHC α*-ketoglutarate dehydrogenase complex, *SuccCoA* succinyl-CoA, *SCS* succinyl-CoA synthesize, *Succ* succinate, *SDH* succinate dehydrogenase, *Fum* fumarate, *Mal* malate, *MDH* malate dehydrogenase, *OAA* oxaloacetate, *CS* citrate synthesize, *Glu* glutamate, *GLS* glutaminase, *GDH* glutamate dehydrogenase

Biochemical assays adopt different strategies for measuring different aspects of a single node of the metabolism. To generalise, the biochemical assays either target a specific metabolite and quantify it or try to decode the enzyme kinetics, one enzyme at a time. The assays targeting the metabolites measure the changes associated with viable (real-time quantification) or non-viable cells (steady-state quantification). Figure 5 shows the points that can be measured by biochemical assays, a list of which is provided in the Supplementary Information (Table S1). These approaches may better reflect changes in metabolite concentrations (Cajka & Fiehn, 2016).

#### Monitoring glutamine metabolism by enzyme kinetics or metabolite quantification assays in a snapshot

Biochemical assays are commonly used due to their affordability, reproducibility, and simplicity (Majdinasab et al., 2021). Glutamine metabolism is closely linked to mitochondrial GLS, which converts glutamine to glutamate through a deamination reaction. This allows glutamate to enter the TCA cycle, generate ATP and NADPH, regulate GSH homeostasis, and control ROS, as shown in Fig. 2. Measuring intracellular levels of substrates such as glutamate, α-KG, ATP, and GLS activity at different time points can provide insight into glutamine metabolism (Leippe et al., 2017). Various colorimetric and fluorometric detection assays are available to measure changes in the aforementioned enzymes or metabolites in the cell (Fu et al., 2017). However, changes in metabolite concentrations are not a reliable indicator of metabolic rates or changes in directionality since an increase in metabolite concentration could signify an increase in precursor enzyme activity and/or a decrease in successor enzyme activity (Bennett et al., 2009; Fendt et al., 2010).

Additionally, biochemical assays are destructive, requiring cells to be lysed at each time point to quantify intracellular metabolites. Therefore, these approaches do not provide continuous kinetic monitoring of glutamine metabolism. However, steady-state approaches based on mathematical modeling are valid during constant exponential growth phase in batch cultures and steady-state in continuous cultures (Buescher et al., 2015; Yasemi & Jolicoeur, 2021). Combining steady-state kinetic monitoring with computational modeling is widely used to study metabolic changes and identify potential therapeutic targets. For instance, a recent study investigated the effect of Bcl-2-associated athanogene 3 (BAG3) on GLS stabilization and reported increased glutaminolysis, as indicated by quantifying glutamate and α-KG levels in cells using appropriate assays (Zhao et al., 2019). The study also suggested BAG3 as an optional therapeutic target against cancer by demonstrating its involvement in the regulation of glutaminolysis (Zhao et al., 2019).

#### Dynamic monitoring of glutamine metabolism by extracellular metabolite quantification assays

To monitor the metabolic activity of a pathway, the real-time viability assay is one of the most widely used tools, which measures the rate of uptake/excretion of metabolites such as glucose, glutamine, lactate, and ammonia, providing an overview of overall cellular metabolism (Antoniewicz, 2013a; Stoll et al., 1996). For example, inhibition of glutamine uptake by l-γ-glutamyl-*p*-nitroanilide (GPNA) (measuring the rate of glutamine degradation from the medium provides information about the rate of glutamine uptake by cells in a culture system) has a dampening effect on the metabolism of gastric cancer cells (Ma et al., 2021).

Furthermore, there are commercially available assays designed to monitor glycolysis flux, which can be used to kinetically monitor glutaminolysis flux as well, because the glutaminolysis pathway leads to lactate production and is hyperactive in proliferating cells. Such assays measure the extracellular acidification rate (ECAR), which is used to quantify proton production and serves as a substitute marker for lactate production (‘Glycolytic rate measurement, glycolysis assay, pH Xtra | Agilent’ n.d.) For instance, Rupprecht et al. determined both ECAR and oxygen consumption rate (OCR) to analyse the influence of glucose and glutamine deficiency on the metabolic activity of N18TG2 cells (Rupprecht et al., 2019). The results showed that glutamine deficiency strongly affected both the OCR and ECAR, leading to their reversal. Glucose deficiency also led to increased glutamine uptake in the cells, indicating that the cells switched to the oxidative metabolism under glucose shortage (Rupprecht et al., 2019). Similarly, Trilla-Fuertes et al. used cell viability assays in combination with computational models to investigate the link between glutaminolysis and breast cancer (Trilla-Fuertes et al., 2020).

Although these assays provided valuable insights, the data alone were not sufficient to fully describe the intracellular fluxes in the metabolic pathway (Junot et al., 2014). Therefore, it is necessary to combine data from these assays, such as the rate of extracellular lactate production, which represents the fluxes of glycolysis and glutaminolysis, with intracellular spectroscopic approaches and physicochemical models to obtain a computational estimate of metabolic fluxes and gain a deeper understanding of cellular metabolism (Buescher et al., 2015; Leippe et al., 2017; Liao et al., 2019).

## Metabolic modelling of glutamine metabolism

The physicochemical modelling approach provides a valuable framework for simulating cellular metabolic kinetics. It helps predict cellular phenotypic responses to external factors such as drugs or changing environmental conditions by providing a quantitative understanding of metabolism and its regulation (Becker et al., 2007), which in turn can generate hypotheses to improve overall process performance by optimising and regulating process conditions and enable potentially useful therapeutic interventions. In addition, experimental validation of these predictions can provide new experimental data that can further improve the models (McGillen et al., 2014; Moulin et al., 2021; Vasilakou et al., 2016)..Because the biological experiments provide the flux information needed to construct the models, accurate identification of metabolites and determination of flux changes is critical to the accuracy of subsequent predictions of network properties made using the models (Acosta et al., 2007). In general, there are different types of mathematical models to describe intracellular reaction processes and metabolism, such as constraint-based models, spatial models, dynamical model components, qualitative and logical models, and kinetic models. For the description of metabolism, most studies have been drawn from these two groups: constraint-based models (or stoichiometric models) and kinetic models (Gombert & Nielsen, 2000; Keating et al., 2020; Nielsen, 2017).

### Constraint based models

**Fig. 6:**
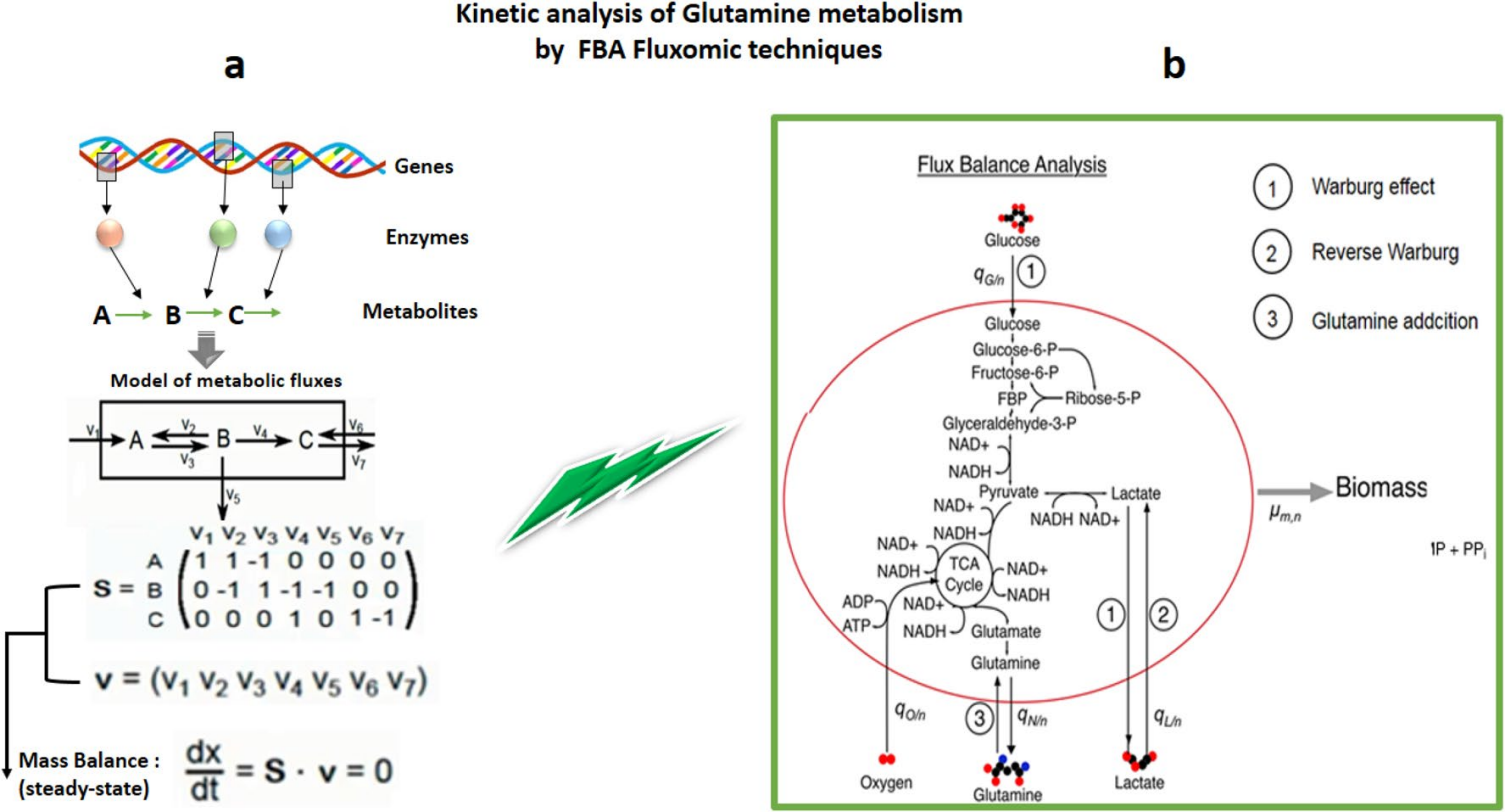
**a** In FBA method, the metabolic reaction fluxes within the boundary can be calculated by solving the equation SV = 0 (S stands for matrix and V stands for reaction rate) using linear programming, optimising or minimising an objective function, such as maximising cell growth, under given constraints. **b** FBA model for central carbon metabolism. The arrows indicate the flux of metabolites in a simplified representation of cell metabolism and growth. The uptake and production rates of the ith metabolite and nth metabolic phenotype are denoted as q_i/n_ [g/g − DW^−hr^]. The FBA model is optimised by imposing the maximum growth rate, μ_m,n_ [hr^−1^], as an objective function for each cell type and metabolic phenotype. **b** Has been adapted from Shan et al. (2018), under the terms of the Creative Commons Attribution License

Constraint-based modelling, also known as stoichiometry-based or FBA, is a mathematical modelling technique for rebuilding a metabolic network model of microorganisms in order to simulate and predict metabolic reaction fluxes, on the basis of which the cellular function can be interpreted (Covert et al., 2001; O’Brien et al., 2015). This technique has been developed based on the knowledge of whole genome sequencing (WGS) of microorganisms and the development of tools for annotating gene functions (Covert et al., 2001; O’Brien et al., 2015). FBA predicts reaction fluxes within defined boundaries of a metabolic network reconstruction based on mass balance or steady-state assumptions, meaning that the metabolite concentration cannot change within the system, e.g. dATP/dt = dNADH/dt = 0, upon which a set of FBA equations can be constructed (Maarleveld et al., 2013; Orth et al., 2010). A summarised form of the FBA process is shown in Fig. 6a. FBA models use the principles of reaction stoichiometry and physicochemical constraints that may include thermodynamics or kinetics such as energy balance, mass balance and flux constraints (Cotten & Reed, 2013). These models use Boolean expressions to represent the relationship between genes, proteins and metabolic reactions (Machado et al., 2016). A popular example of this modelling approach is Genome-scale metabolic models that simulate whole cell metabolic reactions based on genome sequences (using the Human Metabolism Map; Recon2) using the COBRA Toolbox v2.0 library (Ryu et al., 2015). Although, it is hardly possible to build a comprehensive mathematical model for a living cell that can include all cellular processes as many aspects of cellular function are still unknown (Nielsen, 2017), several whole-cell models have been developed, such as a model developed by Markus Covert which represents the most comprehensive mathematical model for a whole cell of *Mycoplasma genitalium* (Karr et al., 2012).

The quality of flux predictions in this technique is significantly affected by the choice of cellular objective. In cancer cells, lactate production as a cellular objective might show the ability to correctly predict high flux through glycolysis pathway (Schwartz et al., 2015). Zhang and Boley (2022) constructed a non-linear multi-objective FBA model in which an objective function contains three important objective terms, namely: ATP production rate, ATP yield and lactate production rate. Using this model, they were able to show the inducing effect of glutaminolysis on the Warburg effect in cancer cells (Zhang & Boley, 2022). A good example of a FBA model created by Shan et al. for central carbon metabolism is shown in Fig. 6b. The model includes labels for the primary steps of the three hypotheses, which are: (1) the Warburg effect, (2) the Reverse Warburg effect, and (3) Glutamine addiction (Shan et al., 2018). Constraint-based models have provided the fundamental biological insights into metabolic pathways (Xu et al., 2013). For example, Damiani et al. (2017) employed the model of the glucose and glutamine metabolism and reported that, when oxygen conditions are insufficient to fully oxidise the available glucose and glutamine carbons (as in the environment of proliferating cells), redox homeostasis can be maintained through switching on alternative metabolic pathways, like utilising glutamine through reductive carboxylation which results in lipid production. Nevertheless, such models do not provide the level of kinetic details of the metabolic pathway that is required to show how changes in the dynamic concentration of cellular components affect functional behaviour (Srinivasan et al., 2015). In recent years, efforts have been made to apply dynamic control of metabolism on the constraint-based approaches by taking extracellular by-products into account, leading to dynamic FBA (dFBA), through which dynamic models to study a bio-system under transient conditions can be developed to capture the dynamic changes in metabolic fluxes through time series of concentration measurements (Llaneras et al., 2012; Yasemi & Jolicoeur, 2021). Dynamic flux analysis approaches are now increasingly applied to investigate and optimise microbial and mammalian bioprocesses (Antoniewicz, 2013b).

### Kinetic models

Kinetic models that express how changes in metabolite concentrations affect local reaction rates are able to dynamically analyse the metabolic state of biological systems and also provide dynamic simulation of metabolism by implementing reaction kinetics (Smallbone & Mendes, 2013). These models require the determination of macroscopic data to obtain a general formulation of the dynamics of the metabolic process (Richelle & Bogaerts, 2015). However, the selection of appropriate model structures is often based on a trial-and-error approach (Richelle & Bogaerts, 2015).

In 1988, Savageau proposed a general kinetic formalism to represent any non-linear function using power equations (S and Volterra Systems, generalised mass actions) (Savageau, 1988). However, these forms were developed to describe the overall reaction rates of a biological network and are not able to describe a double component effect (Richelle & Bogaerts, 2015). Furthermore, parameter estimation in the context of kinetic structure identification is a rather difficult task, as these functions often involve a large number of highly correlated parameters (Richelle & Bogaerts, 2015). Against this background, Haag et al. have proposed an extension of Michaelis–Menten kinetics using the classical extended Monod law to circumvent the problems of identifiability associated with possible over parameterisation (Haag et al., 2003). This general formalism allows for either activation or inhibition effects with a reduced number of parameters. Furthermore, enzyme kinetics are very well illustrated by the Michaelis–Menten equation, which essentially describes the kinetics of an enzyme with two key parameters: the limiting rate (V) and the Michaelis–Menten constant (K_**M**_) (Schnell, 2014). Notably, Karry et al. have recently proposed the “inverse Michaelis–Menten equation”, which is intended for kinetic analyses of enzyme reactions at interfaces, especially for enzyme kinetics in steady state, in which the enzyme concentration far exceeds the molar concentration of the available surface sites (Kari et al., 2017).

**Scheme 1:**
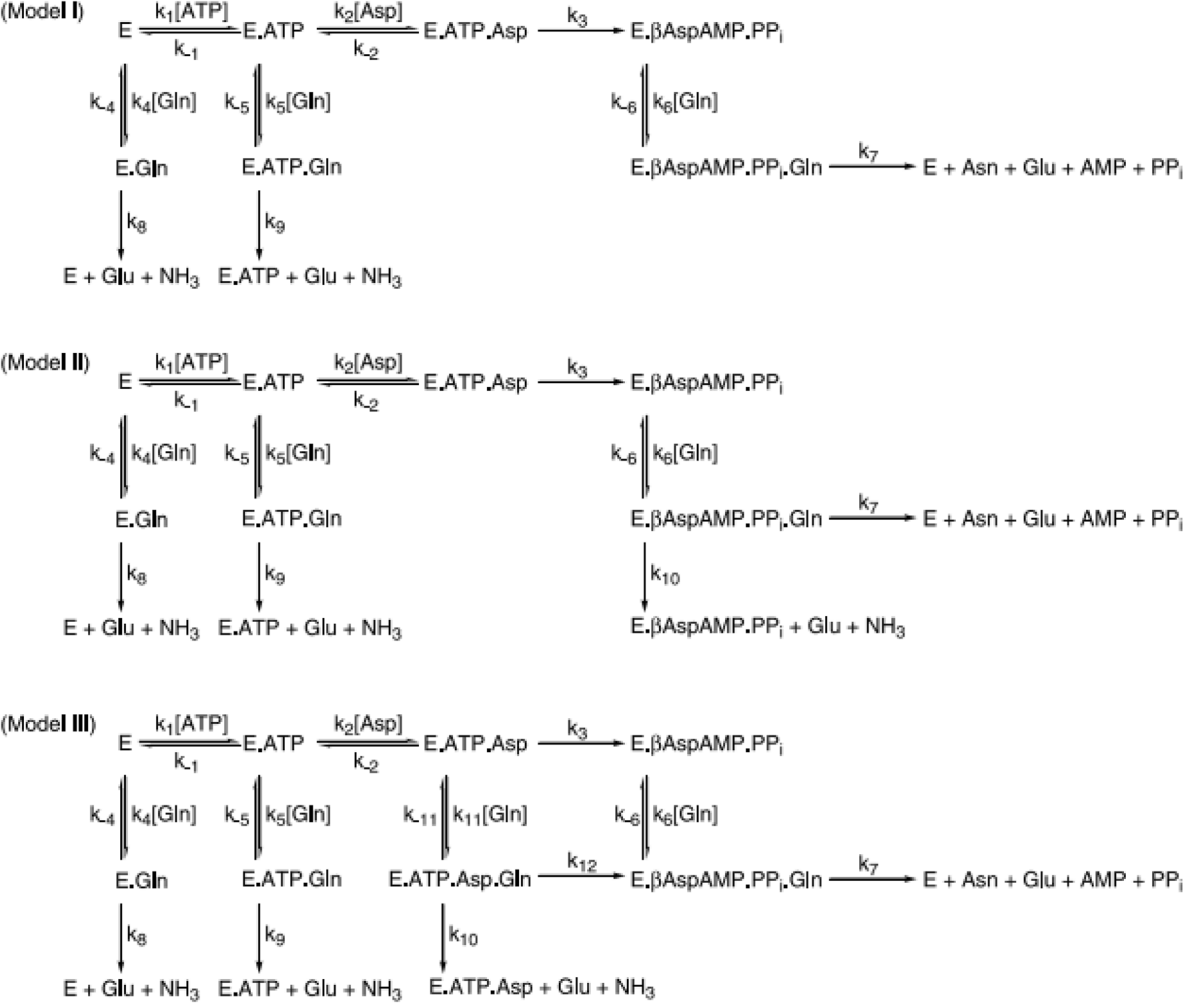
Models for the kinetic mechanism of *Escherichia coli* asparagine synthetase B. (Top) Proposed kinetic mechanism based on product inhibition and isotope trapping experiments (model I) (Middle). Model I modified to include the leakage of ammonia from the intramolecular tunnel (model II). (Bottom) Modified proposal for the AS-B kinetic mechanism that assumes catalytic site coordination after βAspAMP formation and no leakage of ammonia from the intramolecular tunnel (model III). The models are reproduced from Tesson et al. (2003) with permission

There have been various proposed kinetic models for the metabolic pathway of glutamine (Schuster et al., 2021; Wang et al., 2022). The example shown in Scheme 1 examined the kinetic behaviour of glutamine-dependent asparagine synthetases (AS-B) (Tesson et al., 2003). This model was able to demonstrate that the coordination of the two active sites in AS-B occurs only after the formation of a b-aspartyl AMP intermediate in the synthetase site of the enzyme by incorporating a quaternary E.ATP.Asp.Gln complex. This complex can either proceed to the formation of asparagine or release ammonia by unproductive glutamine hydrolysis (Tesson et al., 2003). Another recent study revealed a new role for glutamine as a major source of acetyl-CoA to revive TCA cycle metabolism, which the authors hypothesised could occur under the condition of mitochondrial dysfunction, such as mutations in the ETC or TCA cycle, by which α-KG (from glutamine) is carboxylated by NADPH-dependent isoforms of isocitrate dehydrogenase to produce isocitrate and then acetyl-CoA (Hensley et al., 2013).

The development of the standardised platforms of Systems Biology Markup Language (SBML; Hucka et al., 2003) and Systems Biology Graphical Notation (SBGN; Mi et al., 2009) has led to the emergence of a growing number of open-source computational modelling tools such as Cell Designer (Funahashi et al., 2008), which allows users to easily draw and kinetically model biochemical pathways (Le Novère et al., 2006), and provides an interface to many other metabolic pathway enrichment databases (Xia et al., 2010). Many metabolic models are available in SMBL, along with a MATLAB (The MathWorks, Inc.) script for extracting annotation information (reaction equations) from the corresponding author (Quek et al., 2010). These advances have made kinetic modelling of complex cellular mechanisms and pathways significantly more accessible in recent years (Strutz et al., 2019).

## Future perspectives

Cells constantly adjust their metabolic fluxes pathway preferences to respond to any change in the environment in vivo, and probably the same mechanisms control the behaviour of cells in in vitro culture. Therefore, monitoring the kinetic evolution of metabolic processes in living cells is of great value in the quest to understand physiological responses to modulators (like drugs and signalling molecules)and the early onset of disease (Klein et al., 2022). However, intracellular metabolism is a very complicated network, so it is not possible to assess the overall metabolic behaviour based on the properties of the individual components alone (Volkova et al., 2020). Therefore, to understand cellular metabolic behaviour, it must be analysed holistically by taking advantage of multi omics approaches. Furthermore, in situ monitoring and analysis of cellular metabolism should be both systematic and non-destructive in order to gain a deeper understanding of the kinetics and regulatory mechanisms underlying the metabolic network.

Among the techniques recently developed for real-time monitoring of metabolism, vibrational spectroscopies, including IR and Raman spectroscopy, have attracted much attention because they provide rapid and simultaneous data on multiple variables in a non-destructive manner (Moros et al., 2010). A molecular compound consisting of n atoms has 3n − 6 vibrational modes (3n − 5 for linear molecules). Each vibrational mode of a molecule has a specific band pattern in the spectrum of IR absorption or Raman scattering, and occurs at a specific wavenumber related to its transition energy. Therefore, molecular labelling is not required in vibrational spectroscopic techniques because all sample molecules have specific band patterns, or a “fingerprint”. In complex multicomponent samples, the combined chemical fingerprint can be used to characterise the state of the sample (Sato et al., 2018). This advantage makes it unnecessary to lyse, stain or fix the living sample (Sato et al., 2018). Also, the direct immersion of microscope objectives or fibre-optic probes in the culture medium enables the use of these light-based spectroscopic techniques for in situ or in-line monitoring of important parameters (Lourenço et al., 2012). Both techniques, IR absorption and Raman scattering, have been explored as an integral part of Process Analytical Technologies (PAT), in which, according to the Quality by Design paradigm (Food and Drug Administration, 2004), in situ monitoring of a bioprocess, such as metabolism, can minimise the risk of contamination during sampling and the delays (between sampling and analysis time) associated with off-line methods (Bhatia et al., 2018; Graf et al., 2022), and both techniques have been demonstrated to provide very accurate prediction of the concentrations of various compounds in the cell culture medium (Bhatia et al., 2018; Sellick et al., 2010). IR absorption spectroscopy analyses the amount of absorbed infrared light according to the interaction with a molecule that occurs at a certain wavenumber. It is a popular technique, because it affords rapid sampling and operational simplicity (Beć et al., 2020). The fundamental vibrational frequencies appear in the mid-IR (~ 500–3500 cm^−1^), while the near-IR region (~ 780 nm to 2500 nm) can be used to measure vibrational overtone and combination modes, which are intrinsically orders of magnitude weaker. The weaker absorption in this region gives rise to greater sampling depths, which makes the technique of near-IR spectroscopy popular in, for example, food science (‘Near-Infrared Spectroscopy in Food Science and Technology—Google Books’ n.d.). Mid-IR absorption spectroscopy is well established in analytical chemistry, as a first port of call for materials characterisation and identification and has increasingly found applications in, amongst others, the pharmaceutical sector (Bunaciu et al., 2010). Water is a notoriously strong absorber in the mid-IR, however, and so applications of analysis in aqueous environments are limited, although there have been extensive explorations of biomedical applications of IR based spectroscopic techniques for label free analysis and classification of, for example, (dried) biofluid (Theakstone et al., 2021) and histopathological biopsies (Paraskevaidi et al., 2021). Many studies have used near-NIR and mid-IR spectroscopy to monitor various intracellular metabolic processes, a list of these studies is included in the review paper by Landgrebe et al. (2010). One example is the use of near-IR spectroscopy by Rhiel et al. (2004) for the off-line and real-time monitoring of PC-3 human prostate cancer cells to quantify the consumption rate of glucose and glutamine, which were determined to be 6.8 and 1.8 × 10^−17^ mol/cell s, respectively (Rhiel et al., 2004). This, and similar works, demonstrate the potential of the technique for online, in situ, real time process monitoring, although there remain some challenges related to these instruments, such as the interpretation of the spectral data, which is difficult due to the overlap of the detected peaks (Abu-Absi et al., 2011; Arnold et al., 2003; Scarff et al., 2008). The technique is also limited in the quantification of intracellular glutamine, as the accurate detection range is above the glutamine level in a mammalian cell culture medium (Foley et al., 2012). Compared to near-IR, mid-IR is known to be a more sensitive technique, and its ability to effectively monitor multiple analytes in a bioprocess in real-time has been confirmed by many studies (Roychoudhury et al., 2006), one of which is the work of Rhiel et al., who successfully used Fourier transform mid-IR spectroscopy for real-time and in situ monitoring of glucose and lactate analytes in bioreactor cultures of Chinese hamster ovary for long-term monitoring at industrial production scale (Rhiel et al., 2002). Although the mid-IR region provides more distinct spectral peaks compared to near-IR, the peaks detected from water in aqueous systems such as biological cells (since most part of the cells are composed of water) interfere greatly by overlapping or masking the peaks of other organic molecules and make the analysis complicated (Abu-Absi et al., 2011).

Often considered a complementary technique to IR absorption (‘Biomedical Applications of Synchrotron Infrared Microspectroscopy: A practical approach—Google Books’ n.d.), Raman spectroscopy quantifies the intensity of inelastically scattered monochromatic light by a molecule at varying frequencies. The frequency shift is due to the change in energy of the photon (which corresponds to the energy of a molecular vibration) as it couples to the vibrations of the molecule (Locke et al., 2019). Raman spectroscopy is particularly sensitive to symmetric, polarisable vibrations (e.g. C=C bonds), whereas asymmetric, polar vibrations (e.g. OH) are very strong IR absorbers. Raman can thus be more readily applied to aqueous systems, and is considered a strong candidate for in vivo biomedical applications (Baker et al., 2018). It should be noted that, according to the report by Li et al., Raman spectroscopy performs better than near-IR spectroscopy in terms of accuracy, selectivity, sensitivity and detection range (Li et al., 2018). Furthermore, because Raman spectroscopy can be performed at source wavelengths in the visible or infrared wavelengths, operation in a confocal microscopic mode has led to increasing application for characterisation and monitoring processes at a cellular and subcellular level, with the prospects of high content spectroscopic analysis (Efeoglu et al., 2018; Farhane et al., 2018; McIntyre et al., 2018).

The potential of Raman spectroscopy for real-time in situ monitoring of key metabolic parameters such as glucose, glutamine and lactate in mammalian cell cultures has been demonstrated (Abu-Absi et al., 2011; Santos et al., 2018). Konorov et al. (2012) investigated the autophagic response to starvation stimuli (glutamine deficiency) in living cells using Raman spectroscopy. Raman spectroscopy was able to non-invasively monitor the dynamics of relevant changes through the starvation state and recovery pathway, and they used fluorescence microscopy with GFP-LC3 labelled cells to confirm the obtained results about generated response in the cells. The results show that glutamine deficiency in both cell lines, MCF-7 (human breast cancer cells) and LMD (mouse metastatic prostate cancer cells), leads to a 2.9-fold and 2.1-fold increase in the autophagosomal compartments, respectively (Konorov et al., 2012). The study also identified the 718 cm^−1^ Raman band, related to phospholipids, as a spectral marker associated with the response, as it has a much higher intensity in cells lacking glutamine (Konorov et al., 2012).

**Fig. 7:**
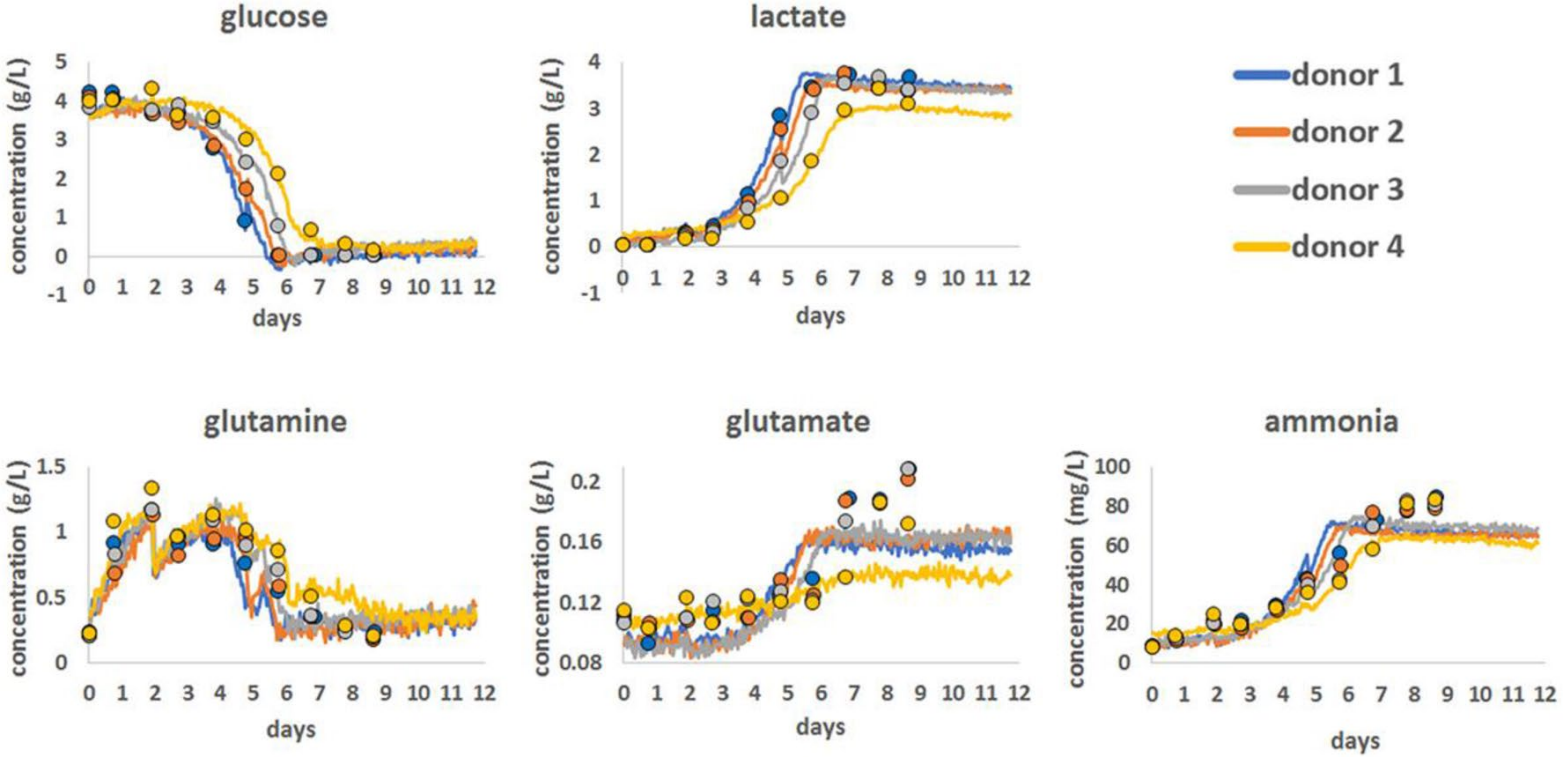
The correlation between chemometric models for glucose, lactate, glutamine, glutamate, and ammonia with reference bioanalyser measurements collected every 24 h, as well as continuous Raman chemometric analysis. The information pertains to a single bioprocess run involving all four donors, and closed circles in the graph represent the reference bioanalyser measurements. Reproduced from Baradez et al. (2018) under the Creative Commons Attribution License

Although the study of Konorov et al. was innovative in its use of Raman microspectrocopy to monitor the metabolic processes in live cell, the spectral analysis was largely qualitative, or univariate, based on the analysis of individual peaks or peak ratios (Konorov et al., 2012). As biological cells are made up of complex molecules such as proteins and DNA, the Raman spectrum becomes very complex, limiting the ability to identify or analyse a particular molecule due to the overlap of bands (Li et al., 2018). Label-free techniques inherently detect all species present within the sampled area, and identification of specific responses requires multivariate analytical methods to extract the differential signatures that are indicative of cellular injury events or response pathways. These signatures can be influenced by changes in concentration or conformation of any or all constituent biomolecules, as well as environmental factors such as pH. It is becoming increasingly clear that approaches focused on identifying differential spectroscopic signatures, rather than specific bands associated with individual biomolecules, may be more effective. As a result, chemometric or multivariate data analysis tools are necessary for extracting the desired information. These techniques help to extract the quantitative concentration information to facilitate the interpretation of the spectral data by solving problems such as background fluctuations, baseline drift or fluorescence (Bos et al., 2020; O’Connell et al., 2010). In other words, the precision of Raman performance depends on chemometric models or multivariate analyses to process the large dataset consisting of thousands of variables and to identify the relationship between the variables (Yousefi-Darani et al., 2022). In unsupervised analyses, a Principal Components Analysis approach is commonly employed to reduce the dimensionality of the data, and in doing so identify the main spectral features which give rise to variances or between datasets. Systematic and/or evolving sources of variance are better explored, or data mined, using regression or correlation analyses (Byrne et al., 2020). The study by Baradez et al. explored Raman spectroscopy as a tool for in-line monitoring of an autologous T-cell immunotherapy model produced in a stirred tank bioreactor system (Baradez et al., 2018). Chemometric models were constructed based on multivariate regression (Projection to Latent Structures) of the Raman spectral data generated by continuous in line monitoring of the bioreactor against off-line reference data for glucose, lactate, glutamine, glutamate, and ammonia concentrations, with reference also to Raman spectra of the pure components. Figure 7 demonstrates that the multivariate regression approach was able to generate models for real time, in situ monitoring of the constituent components.

The ability of Raman microspectroscopy to monitor the uptake of chemotherapeutic agents such as Doxorubicin (DOX) in in A549 and Calu-1 lung cancer cells has been demonstrated (Farhane et al., 2015a, 2017a). Partial Least Squares Regression (PLSR) was furthermore explored to differentiate between the spectral signatures of the initial intracellular chemical binding of cisplatin (Nawaz et al., 2010, 2011), vincristine (Nawaz et al., 2013) and more recently DOX (Farhane et al., 2015b, 2017a, b; Szafraniec et al., 2016) and Actinomycin (Farhane et al., 2018a) from the subsequent cellular response pathway. 2D Spectral Cross-Correlation has been employed to similarly differentiate between drug binding events and cellular response pathways (Farhane et al., 2018b), and Spectral Cross-Correlation in cellular maps has been demonstrated as a sensitive tool to profile the subcellular distribution of specific spectral signatures (Keating et al., 2012). Another flexible chemometric approach is Multivariate Curve Resolution-Alternating Least Squares (MCR-ALS) analysis, which can be used to data mine spectra which are representative of the time course of a bioprocess and interpret changes in the evolution of the chemical components of the process (Jaumot et al., 2005; Pérez-Guaita et al., 2022). Koch et al. have demonstrated the use of MCR-ALS for extracting data on changes in multiple analytes in a bioprocess measured by FT-IR spectroscopy (Koch et al., 2014). However, chemometric techniques require the isolation of information about the system to avoid abstruseness (Pérez-Guaita et al., 2022). The performance of the MCR-ALS approach is guided by constraints, which ensure, for example, non-negativity of the component spectra (de Juan & Tauler, 2021), and were extended to include kinetic constraints, in a Hard–Soft modelling approach, such that the analysis of the evolution of a series of measurements can constrained to fit a kinetic model (De Juan et al., 2000). Such an approach has been employed, for example, by Perez-Guaita et al. to data mine the Raman spectroscopic signatures of the cellular uptake of and response to DOX (Perez-Guaita et al., 2020, 2022).

**Fig. 8:**
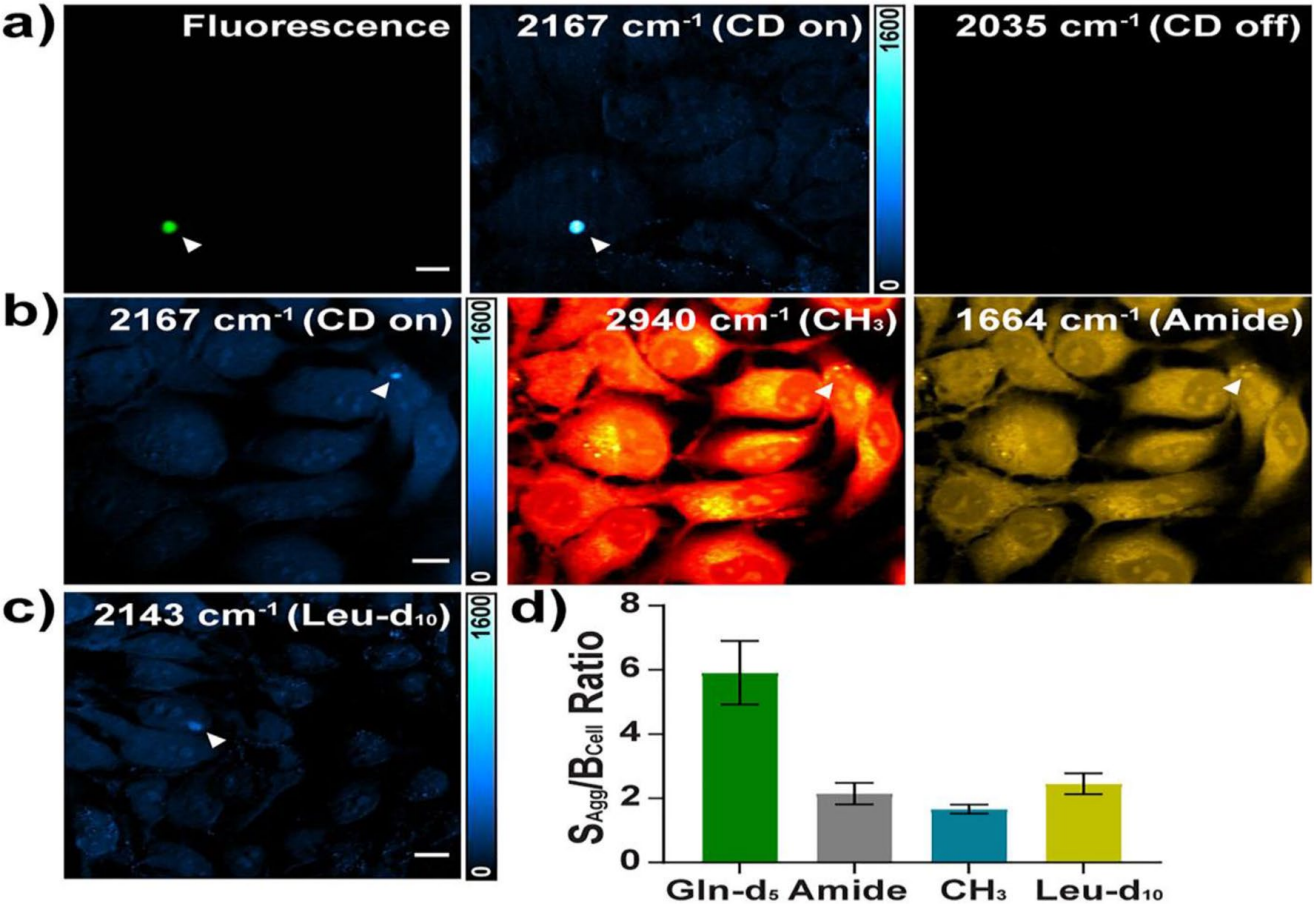
Live-cell SRS imaging of mHtt-97Q-GFP aggregates with Gln-d5 labelling. **a** SRS imaging of mHtt aggregates (arrowheaded, 2167 cm^−1^, C–D on), validated by fluorescence imaging through GFP (Fluorescence). Off-resonance image at 2035 cm^−1^ shows no signal. **b** Live-cell SRS images for an mHtt-97Q-GFP aggregate (arrowheaded) at Gln-d5 (2167 cm^−1^), CH_3_ (2940 cm^−1^), and amide I (1664 cm^−1^) channels on the same set of HeLa cells. **c** SRS imaging of an mHtt-97Q-GFP aggregate at 2143 cm^−1^ by leucine-d10 (Leu-d10) labelling. **d** Average Sagg/Bcell from SRS images of C–D with Gln-d5 labelling (5.75 ± 1.03, n = 13); amide I (2.15 ± 0.34, n = 4); CH_3_ (1.66 ± 0.14, n = 10); and C–D with Leu-d10 labelling (2.45 ± 0.33, n = 10). *Error bar* SD. Reproduced from Miao and Wei (2020) under the terms of the Creative Commons Attribution License

The studies to date illustrate that the continued development of protocols for harnessing and interpreting the label free, high content spectral information which Raman microspectroscopic analysis can yield is imperative, and this is further driven by the demands of emerging technologies. Although full spectral coherent Raman microspectroscopic imaging is currently only available in limited frequency ranges, it has already been demonstrated (Camp et al., 2014), and this imaging technique holds great promise for label-free high-content spectroscopic analysis of sub-cellular processes in real-time, as well as unparalleled visualization of cellular processes and function.. In this context, a recent live cell imaging study proposed a new platform for combining stimulated Raman (SRS) with deuterium-labelled glutamine (Gln-d5) to analyse the nature of polyglutamine (polyQ) aggregates in Huntington's disease (Miao & Wei, 2020). They achieved high sensitivity and specific Raman imaging of aggregated Gln-d5 in living cells (Fig. 8), which could lead to the conclusion that (1) these aggregates are 1.8 times denser compared to those with GFP labelling used in fluorescence microscopy, (2) the composition of the proteins depends on the size of the aggregates and (3) the inhibition of aggregate formation reveals the possibility of an intermediate folding state caused by local hyperhydration of the aggregates (the associated resulting data indicate higher C–H/C–D binding ratios favouring the possibility of interaction with heat shock protein (HSP) 40/70) (Miao & Wei, 2020).

**Fig. 9:**
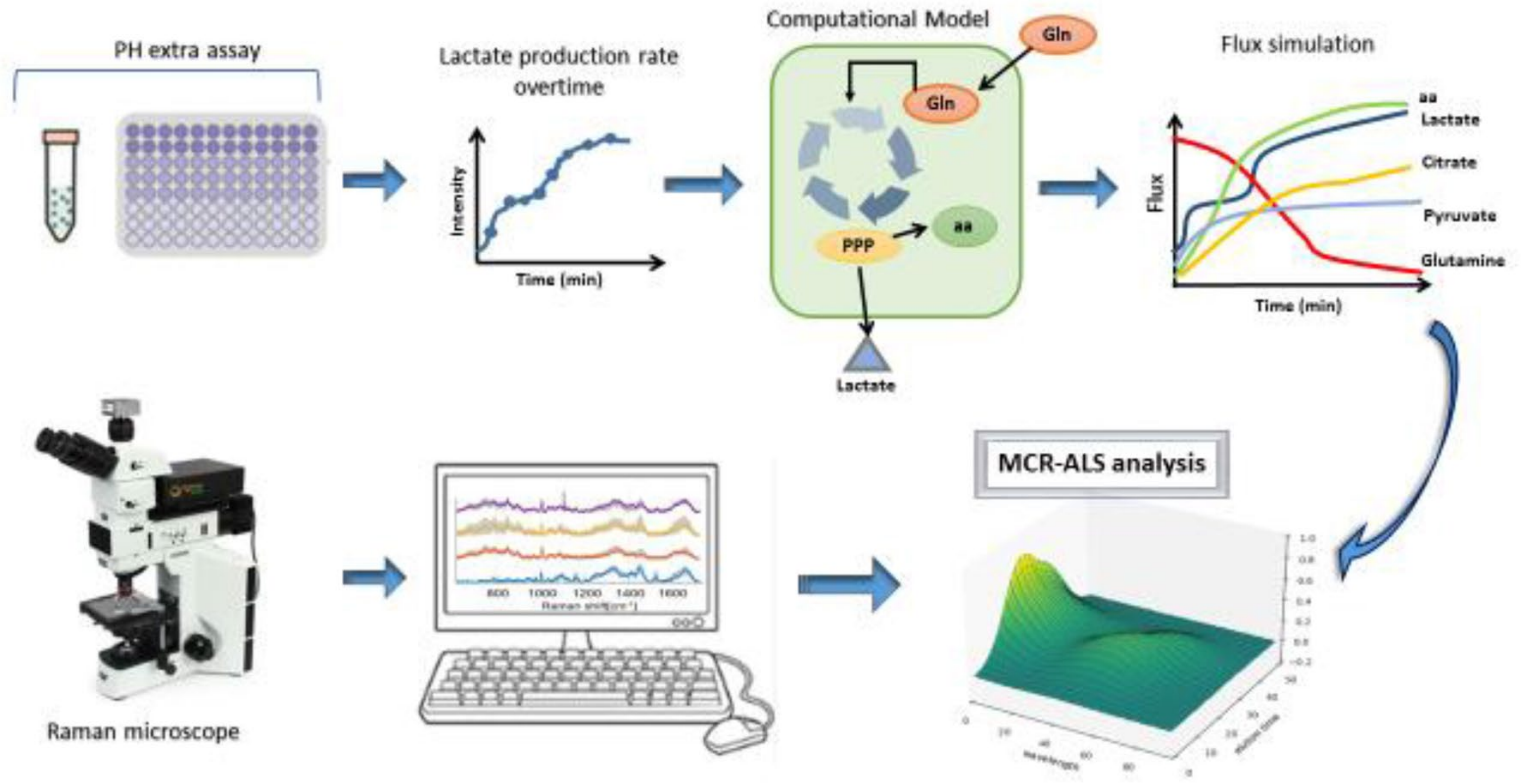
Schematic illustration of proposed paradigm of use of conventional biochemical assays on which to develop metabolic flus models, which can in turn be used to guide the multivariate analysis of label free, live cell Raman microspectroscopic data

Reviewing the methods used to monitor glutamine metabolism, it can be concluded that Raman spectroscopy has more desirable properties as a quantitative analytical approach compared to conventional analytical methods such as MS or NMR. Label-free monitoring using Raman microspectroscopy not only avoids bias in biological response, but is also capable of non-invasively monitoring the metabolic dynamics of living organisms to develop more effective metabolic intervention strategies for therapeutic approaches (Konorov et al., 2012; Lussier et al., 2019). Moreover, extracellular metabolites can be simultaneously analysed with Raman (Shalabaeva et al., 2017). Therefore, for real-time and in situ monitoring the cellular kinetics of metabolic pathway of glutaminolysis, it is conceivable to employ kinetic models, informed by time series, conventional biochemical models such as the pH extracellular assay (measures the lactate production rate), as the basis on which multivariate data mining techniques such as MCR-ALS can reliably extract the signatures of interest from label-free, Raman microspectroscopic analysis of live cells, and interpret them in terms of their biochemical origin. (Fig. 9).

## Supplementary Information

Below is the link to the electronic supplementary material.

(DOCX 33 kb)

## Data Availability

For more detailed data, please refer to the supplementary material of the referenced paper.
