# Supplementary material for: Monitoring and modelling the glutamine metabolic pathway: a review and future perspectives
Source: Metabolomics. 2023 Jul 23;19(8):67. doi: 10.1007/s11306-023-02031-9 (PMC10363518; doi:10.1007/s11306-023-02031-9)
Supplement: Supplementary file 1 — (DOCX 33 kb) [file 11306_2023_2031_MOESM1_ESM.docx]

***Monitoring & Modelling the Glutamine metabolic pathway: A review***

Zohreh Mirveis^1,2^*, Orla Howe^3^, Paul Cahill^4^, Nitin Patil^1,2^, Hugh J. Byrne^1^

*^1^FOCAS Research Institute, Technological University Dublin, City Campus, Camden Row, Dublin 8, Ireland*

*^2^School of Physics and Optometric & Clinical Sciences, Technological University Dublin, City Campus, Grangegorman, Dublin 7, Ireland*

*^3^School of Biological and Health Sciences, Technological University Dublin, City Campus, Grangegorman, Dublin 7, Ireland*

*^4^School of Biotechnology, Dublin City University, Glasnevin, Dublin 9, Ireland.*

**Corresponding Author:* [D21127294@mytudublin.ie](mailto:D21127294@mytudublin.ie)

***Supplementary Information***

**Table S1** List of biochemical assays (colorimetric, fluorometric and luminescent) used to measure metabolite concentrations and enzyme activities in glutamine metabolic pathways.

| Assay Number | **Assay Name** | **Detection Method** | **Detection Limit** | **Platform** | **Company** |
| --- | --- | --- | --- | --- | --- |
| 1 | Pyruvate Colorimetric/Fluorometric Assay Kit | Absorbance (570 nm) or Fluorescence (Ex/Em 535/587 nm) | 1 µM to 10 mM pyruvate. | Microplate reader | Abcam |
| 2 | Acetyl-CoA Fluorometric Assay Kit | Fluorescence (Ex/Em 535/587 nm) | 10 to 1000 p mol of Acetyl CoA (with detection limit ~0.4 μM) | Microplate reader | Abcam |
| 3 | Coenzyme A (CoA) Colorimetric/Fluorometric Assay Kit | Absorbance (570 nm) or Fluorescence (Ex/Em 535/587 nm) | detect 0.1 to 10 nmol of CoA (2.5-250 µM concentration range) | Microplate reader | Abcam |
| 4 | Citrate Synthase Activity Colorimetric Assay Kit | Absorbance (412 nm) | detect Its activity less than 1 mU | Microplate reader | Abcam |
|  | mtCheck™ Citrate Synthase Assay Kit | absorbance at 412 nm |  | Spectrophotometry | Creative Biogene |
| 5 | mtCheck™ Mitochondrial Citric Acid Assay Kit | absorption at 330nm |  | Spectrophotometry | Creative Biogene |
|  | Citrate Colorimetric/Fluorometric Assay Kit | Absorbance (570 nm) or Fluorescence (Ex/Em 535/587 nm) | detect 0.1 to 10 nmoles (~2 µM-10 mM) of citrate | Microplate reader | Abcam |
| 6 | mtCheck™ Aconitase Activity Assay Kit | absorption at 340nm |  | Spectrophotometry | Creative Biogene |
|  | mtCheck™ Aconitase Activity Assay Kit |  |  | Microplate reader | Creative Biogene |
|  | Aconitase Activity Colorimetric Assay Kit | Absorbance at 450 nm |  | Microplate reader | Abcam |
| 7 | Isocitrate Colorimetric Assay Kit | Absorbance at 450 nm | detect 1 to 20 nmoles (~0.2 – 5 µg) of isocitrate. | Microplate reader | Abcam |
| 8 | Isocitrate Dehydrogenase Activity Colorimetric Assay Kit | Absorbance at 450 nm | detect as low as 0.01 mU | Microplate reader | Abcam |
|  | mtCheck™ Mitochondrial Isocitrate Dehydrogenase Assay Kit | absorption at 340nm |  | Spectrophotometry | Creative Biogene |
| 9 | Alpha-Ketoglutarate Colorimetric/Fluorometric Assay Kit | Absorbance (570 nm) or Fluorescence (Ex/Em 535/587 nm) | detect in the range of 0.01 to 10 n moles | Microplate reader | Abcam |
| 10 | mtCheck™ Alpha-ketoglutarate Dehydrogenase Assay Kit | absorption at 340nm |  | Spectrophotometry | Creative Biogene |
|  | Alpha-Ketoglutarate Dehydrogenase Activity Colorimetric Assay Kit | Absorbance at 450 nm | detect less than 0.1 mU | Microplate reader | Abcam |
| 11 | Succinyl-CoA Synthetase Activity Colorimetric Assay Kit | Absorbance at 450 nm | detect less than 0.1 mU | Microplate reader | Abcam |
| 12 | Phosphate Assay Kit (Fluorometric) | Fluorescence (Ex/Em 535/587nm) | 2 µM - 10 µM | Microplate reader | Abcam |
|  | Phosphate Assay Kit (Colorimetric) | Absorbance (650 nm) | 0.001 mM - 1 mM | Microplate reader | Abcam |
|  | Phosphate Assay Kit (Fluorometric) | Fluorescence (Ex/Em 535/587nm) | 0.1 nmol/well - 0.5 nmol/well | Microplate reader | Abcam |
| 13 | Succinate Assay Kit (Colorimetric) | Absorbance at 450 nm | 40 µM | Microplate reader | Abcam |
| 14 | Succinate Dehydrogenase Activity Colorimetric Assay Kit | Absorbance (OD = 600 nm) | detect less than 0.1mU | Microplate reader | Abcam |
| 15 | FAD Colorimetric/Fluorometric Assay Kit | Absorbance (570 nm) or Fluorescence (Ex/Em 535/587 nm) | detect less than 1 nM FAD. | Microplate reader | Abcam |
| 16 | Fumarate Colorimetric Assay Kit | Absorbance at 450 nm | detect as low as 1 nmol of fumarate per well (20 µM). | Microplate reader | Abcam |
| 17 | Fumarase Colorimetric Assay Kit | Absorbance at 450 nm | detect less than 50 U/ml of fumarase | Microplate reader | Abcam |
| 18 | Malate Colorimetric Assay Kit | Absorbance at 450 nm | detect 1 ~ 10 nmol of Malate in a 50 µl sample | Microplate reader | Abcam |
| 19 | Malate Dehydrogenase Activity Colorimetric Assay Kit (detects both isoforms of MDH: MDH1 and MDH2) | Absorbance at 450 nm | detect less than 0.5 mU of MDH | Microplate reader | Abcam |
|  | mtCheck™ Mitochondrial Malate Synthase Assay Kit | absorption at 340nm |  | Spectrophotometry | Creative Biogene |
| 20 | NAD/NADH Quantitation Colorimetric Kit | Absorbance at 450 nm | 400 nM - 2000 nM | Microplate reader | Abcam |
| 21 | Oxaloacetate Colorimetric/Fluorometric Assay Kit | Absorbance (570 nm) or Fluorescence (Ex/Em 535/587 nm) | detect 0.1-10nmol (2-200 µM) of OAA. | Microplate reader | Abcam |
|  |  |  |  |  |  |
| 22 | Glutamine Assay Kit (Colorimetric) | Absorbance | detect as little as 25 μM of glutamine | Microplate reader | Abcam |
| 23 | Ammonia Assay Kit (Colorimetric) | Absorbance (570 nm) | detect 1 nmol (~20 µM) of total ammonia and ammonium | Microplate reader | Abcam  Biovision |
| 24 | Glutamate Dehydrogenase (GDH) Activity Assay Kit  (Colorimetric) | Absorbance (450 nm) | detect GDH activity as low as 0.01 mU | Microplate reader | Merk  AppexBio |
| 25 | Lactate-Glo™ Assay  (bioluminescent) | luminescence | detection range up to 200µM | Microplate reader | Promega |
